# Antimicrobial activity of the methanolic leaf extract of *Prosopis laevigata*

**DOI:** 10.1038/s41598-022-25271-6

**Published:** 2022-12-02

**Authors:** Uriel Nava-Solis, Mario Rodriguez-Canales, Ana Bertha Hernandez-Hernandez, David Arturo Velasco-Melgoza, Brenda Paola Moreno-Guzman, Marco Aurelio Rodriguez-Monroy, María Margarita Canales-Martinez

**Affiliations:** 1 Laboratorio de Farmacognosia, Unidad de Biología, Tecnología Y Prototipos (UBIPRO), Facultad de Estudios Superiores Iztacala, Universidad Nacional Autónoma de México, Av. de los Barrios No. 1, Los Reyes Iztacala, Tlalnepantla, Edo. de México, C.P. 54090 México; 2 Laboratorio de Investigación Biomédica en Productos Naturales, Carrera de Medicina, Facultad de Estudios Superiores Iztacala, Universidad Nacional Autónoma de México, Avenida de los Barrios Numero 1, Colonia Los Reyes Iztacala, Tlalnepantla, Edo. de México, C.P. 54090 México

**Keywords:** Microbiology, Plant sciences, Health care

## Abstract

The appearance of antimicrobial-resistant pathogens has highlighted the need to search for new compounds that can effectively combat infectious diseases. A potential source of these compounds are the secondary metabolites of species that have been reported as effective traditional treatments of such diseases. *Prosopis laevigata* is a medicinal plant, and its chemical constituents have shown potential antimicrobial activity. In this study, the antimicrobial activities of the methanolic extract of the leaves of *Prosopis laevigata* against different bacterial and fungal strains of medical and agronomic interest were investigated in vitro. In addition, the chemical composition of this extract was investigated by HPLC–DAD, GC‒MS, and HPLC‒MS. The methanolic leaf extract contained 67 mg of GAE/g of total phenols (6.7%), 2.6 mg of QE/g of flavonoids (0.26%), and 11.87 mg of AE/g of total alkaloids (1.18%). Phenolic acids and catechol were the compounds identified by HPLC–DAD. The methanolic extract had strong antimicrobial activity, especially against *Staphylococcus aureus* (MIC = 0.62 mg/mL), *Escherichia coli* (MIC = 0.62 mg/mL), *Candida tropicalis* (MIC = 0.08 mg/mL) and *Fusarium moniliforme* (MIC = 4.62 mg/mL). These results suggest that the extract of *P. laevigata* leaves could be a source of antimicrobial molecules. However, it is necessary to delve into its chemical composition.

## Introduction

The loss of effectiveness of antibiotics due to the emergence of resistant pathogens (bacteria, fungi, parasites, etc.) currently represents one of the main challenges that is faced by public health at the global level^1,2^. It is estimated that by the year 2050, infections caused by these types of organisms will be among the main causes of mortality worldwide^3,4^. One of the main research directions to combat this problem has focused on the search for novel molecules that can complement or replace the antibiotics that are currently used and have lost their effectiveness over time^2,4^. A great number of these investigations explore secondary metabolites from fungi, plants, and animals for this purpose, sometimes using traditional medicine as a starting point to identify compounds of interest^5–7^.

One of the main vegetal genera with greater use in traditional medicine that has been investigated in recent years for its antimicrobial activity is *Prosopis*, whose members are popularly known as “mesquites” or “algarrobos” in different regions of America, Africa, and Asia^8^. The bark, leaves, stems, roots, flowers, and fruits from *P. africana, P. alba, P. cineraria, P. farcta, P. glandulosa, P. juliflora, P. nigra, P. spicigera* and *P. laevigata* have been used for the treatment of different infectious diseases, especially cutaneous, respiratory, and digestive conditions^8–10^. In in vitro studies*,* the use of extracts (especially aqueous, methanolic, ethanolic, chloroform, and acetonic extracts) and specific fractions obtained by chromatographic methods from the aerial parts of the species previously mentioned have shown antimicrobial activity against numerous strains of medical interest, such as methicillin-resistant *Staphylococcus aureus* (MRSA), *Escherichia coli, Streptococcus mutans, Shigella flexneri, Listeria monocytogenes* and *Candida albicans*; these activities have been attributed to the secondary metabolites of these plants, especially phenolic acids, flavonoids, and alkaloids^8–10^.

*Prosopis laevigata* is the most widely distributed species of the genus *Prosopis* in the arid zones of northern and central Mexico. The plants have a predominantly arboreal growth, reaching 15 m in height. It has bipinnate leaves and a greenish-yellow inflorescence about 10 cm long. its legume has an approximate size of 12–17 cm long × 1–1.4 cm wide, being yellow in color and with violet longitudinal striated spots; its seeds have an ovate to elliptical outline; Its flowering season is from the end of February to the end of April and its fruiting season is from May to August, and it has a harvest period between August and October. This species is used for forage, obtaining wood, coal, and honey and its fruits are used for the preparation of flour^11,12^; likewise, its bark, leaves, and in general, its aerial part have been reported about their traditional uses against cutaneous, digestive and respiratory diseases, besides in addition to being cardioprotective, antioxidant and antimicrobial activity^8–12^.

The purpose of this study was to evaluate the antimicrobial potential of compounds present in the leaves of *P. laevigata* against different strains of pathogens of medical and agronomic interest.

## Results

### Chemical analysis of the methanolic extract

In qualitative tests, the *P. laevigata* methanolic leaf extract was positive for the presence of phenolic compounds, flavonoids, and alkaloids. When quantifying these groups, it was observed that the extract contained more phenolic compounds than alkaloids; however, the alkaloid content was higher than that of flavonoids. On the other hand, the antioxidant capacity is considered poor according to the antioxidant activity index (AAI = 0.09)^13^ (Table 1).

**Table 1:** Total contents of phenols, flavonoids, and alkaloids in the methanolic extract of *P. laevigata* leaves.

	Qualitative identification	Total content (mg)	Percentage of methanolic extract
Total phenols (mg GAE/g)	+	67.00 ± 0.00	6.7%
Total flavonoids (mg QE/g)	+	2.6 ± 0.00	0.26%
Total alkaloids (mg AE/g)	+	11.87 ± 0.00	1.18%
Antioxidant capacity (ppm)	−	334.78 ± 0.00	–

+ qualitative presence, − no determined.

**Figure 1:**
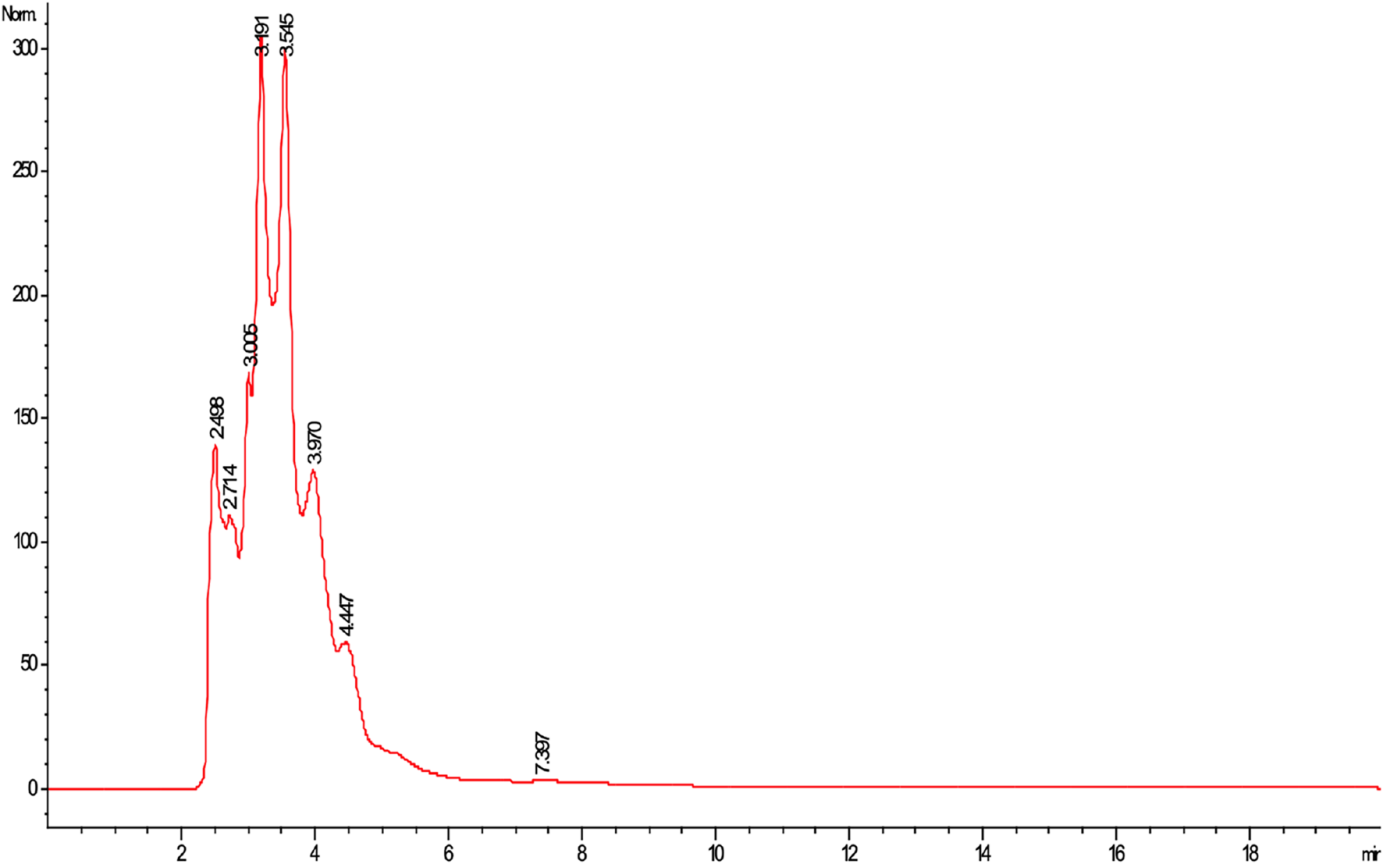
Chromatogram of *Prosopis laevigata* methanolic leaf extract obtained by HPLC–DAD.

Chemical composition analysis by HPLC highlighted some simple phenols (λ_max_ = 220, 270) and the presence of catechol (λ_max_ = 262, retention time 3.981 min.) (Fig. 1). GC‒MS and HPLC‒MS analyses were not conclusive because the retention times, UV spectra, and mass spectra did not match those of our database standards.

### In vitro* antimicrobial activity*

The activity of the methanolic leaf extract against the chosen microbial strains is shown in Table 2. In general, it was observed that its activity against the bacterial strains was similar, with MIC values in the range of 0.62 to 10 mg/mL, and Gram-positive bacteria being on average more sensitive to the methanolic extract than the Gram-negative bacteria, with the exception of *Escherichia coli* clinical case^2^, whose MIC was similar to those observed for the *Staphylococcus spp.* strain, and significant differences were found between the effect of the extract and the positive control for the strains tested (F = 0.0018, p = 0.001).

**Table 2:** Antimicrobial activity of the methanolic extract of *P. laevigata* leaves.

Strain	Inhibition diameter (mm)	Control* (mm)	MIC (mg/mL)	MBC or MFC (mg/mL)
*Staphylococcus aureus* ATCC 12,398	10.00 ± 0.0	14.00 ± 1.00	0.62	1.25
*Staphylococcus aureus* clinical case	6.00 ± 0.58	10.30 ± 0.58	1.25	2.50
*Staphylococcus epidermidis* ATCC 12,228	6.00 ± 0.00	9.30 ± 1.15	5.00	10.00
*Staphylococcus epidermidis* clinical case	10.00 ± 0.58	16.00 ± 4.58	0.62	1.25
*Enterococcus faecalis* ATCC 29,210	8.00 ± 0.58	15.00 ± 1.00	0.62	1.25
*Pseudomonas aeruginosa CDBBB*999	5.00 ± 0.00	10.60 ± 1.58	2.50	5.00
*Escherichia coli* ATCC 25,922	8.00 ± 1.52	6.00 ± 1.00	5.00	10.00
*Escherichia coli* clinical case^1^	5.00 ± 0.00	9.00 ± 1.00	5.00	10.00
*Escherichia coli* clinical case^2^	8.00 ± 0.58	20.30 ± 0.58	0.62	1.25
*Vibrio cholerae* clinical case	10 .00 ± 0.58	19.00 ± 1.00	10.00	20.00
*Candida albicans* ATCC 14,065	11.33 ± 0.57	22.00 ± 1.00	0.04	0.08
*Candida albicans* ATCC 32,354	13.33 ± 0.57	20.33 ± 0.57	0.04	0.08
*Candida albicans* CDBB-L-1003	12.00 ± 0.00	20.00 ± 0.00	0.08	0.16
*Candida albicans* ATCC-10231	10.66 ± 0.57	20.00 ± 0.00	0.16	0.31
*Candida albicans*, clinical case^1^	13.33 ± 1.15	16.50 ± 4.00	0.08	0.16
*Candida albicans,* clinical case^2^	12.00 ± 0.00	18.66 ± 1.15	0.08	0.16
*Candida tropicalis* CDBB-L-1098	13.33 ± 0.57	15.66 ± 0.57	0.08	0.16
*Candida tropicalis* clinical case^1^	16.00 ± 0.00	9.33 ± 0.57	0.08	0.16
*Candida tropicalis,* clinical case^2^	13.00 ± 0.00	20.00 ± 0.00	0.08	0.16
*Candida tropicalis* clinical case^3^	12.00 ± 0.00	20.33 ± 0.57	0.08	0.16
*Candida tropicalis,* clinical case^4^	12.66 ± 0.57	18.66 ± 2.30	0.16	0.63
*Candida glabrata* CBS 138	12.33 ± 0.57	13.33 ± 5.77	0.31	0.63
*Candida glabrata* CDBB-L1536	13.00 ± 0.00	23.33 ± 0.57	0.08	0.16
*Candida glabrata,* clinical case^1^	11.66 ± 0.57	21.33 ± 1.25	0.16	0.31
*Candida glabrata*, clinical case^2^	11.66 ± 0.57	14.00 ± 3.46	0.16	0.31
*Fusarium moniliforme* CDBB-H-265	–	–	4.62	–
*Fusarium subglutinans*	–	–	6.02	–
*Fusarium sporotrichioides ATCC NRLL3299*	–	–	6.10	–
*Fusarium oxysporum*	–	–	6.48	–
*Rhizoctonia lilacina* CDBB-H-306	–	–	7.55	–

*Bacterial positive control: chloramphenicol 25 μg/disk; yeast positive control: nystatin 25 μg/disk. (−): no determined.

In the case of yeasts, the MIC range was smaller than that observed for the bacterial strains, ranging from 0.04 mg/mL to 0.31 mg/mL, with the *Candida albicans* strains on average being the most sensitive to the methanolic extract, and *C. glabrata* strains being the most resistant (Table 2). The comparative effect between nystatin (positive control) and the methanolic extract showed a significant difference (F = 17.06 and p = 0.001). The effect of the methanolic extract was remarkable because it had a higher effect than the positive control.

**Figure 2:**
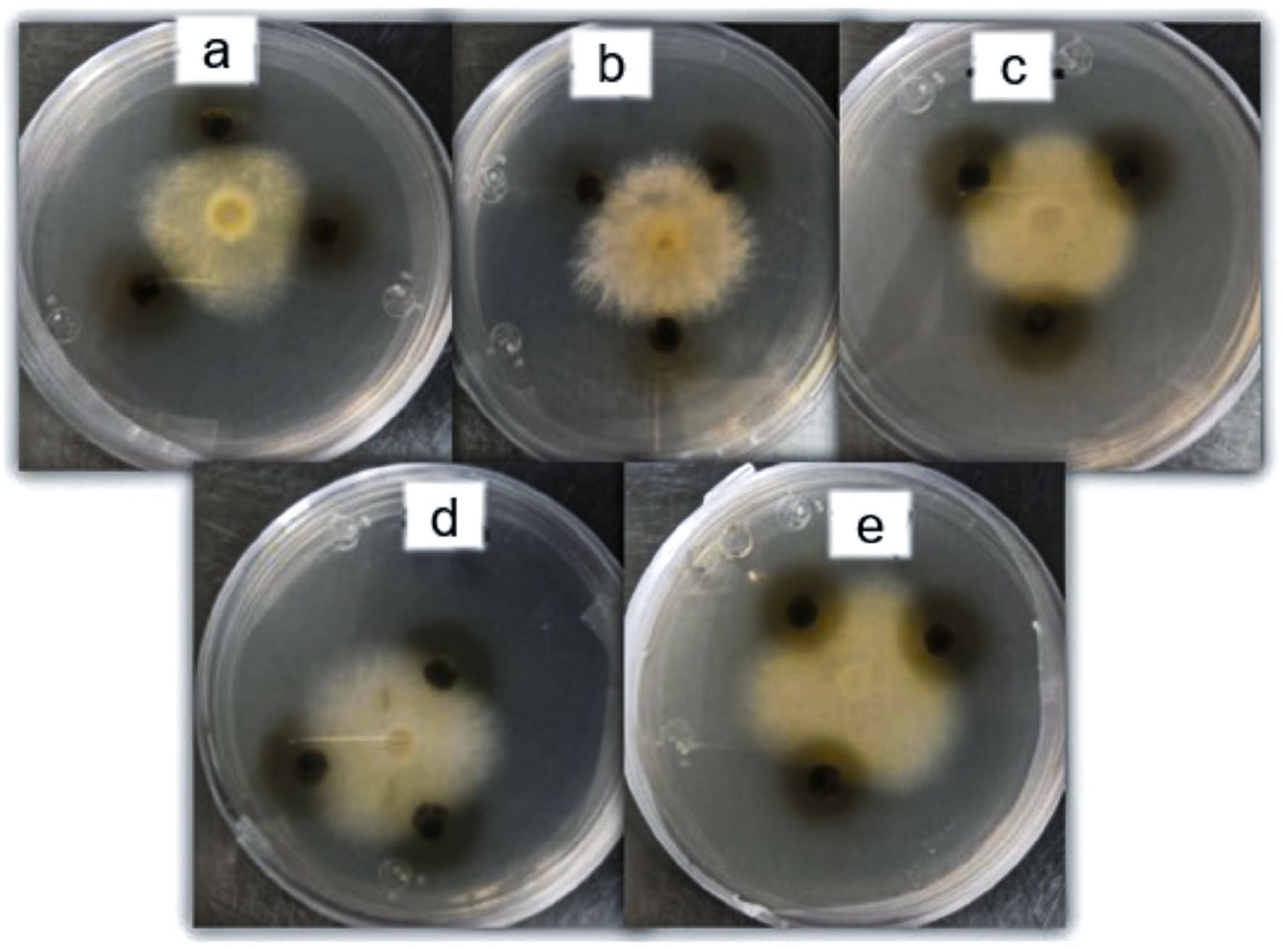
Activity of the *P. laevigata* methanolic leaf extract against some filamentous fungi: (**a**) *Fusarium sporotrichioides* ATCC NRLL3299, (**b**) *Fusarium moniliforme* CDBB-H-265, (**c**) *Fusarium oxysporum*, (**d**) *Fusarium sp.,* and (**e**) *Fusarium subglutinans*.

Finally, the group of filamentous fungi had a higher range of MIC values, from 4.62 to 7.55, with *Fusarium moniliforme* CDBB-H-265 being the most sensitive and *Rhizoctonia lilacina* CDBB-H-306 being the most resistant (Table 2 and Fig. 2).

**Figure 3:**
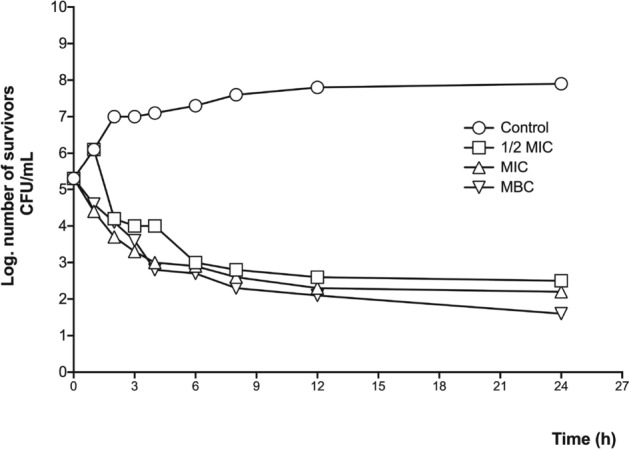
Time-killing bacterial kinetic assay of the methanolic leaf extract against *S. aureus* ATCC 12,398. 1/2 MIC = 0.31 mg/mL; MIC = 0.62 mg/mL; MBC = 1.25 mg/mL.

**Figure 4:**
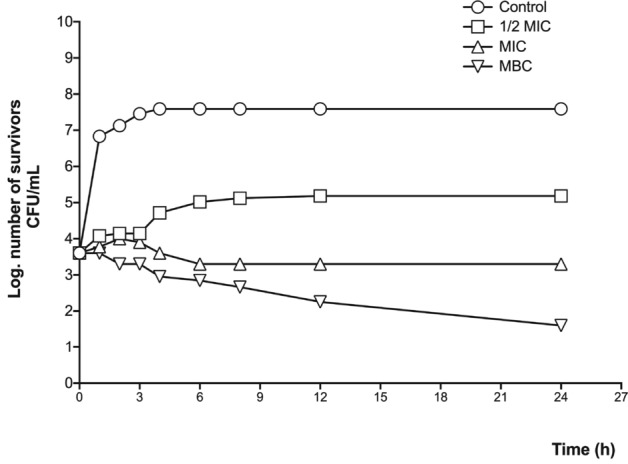
Time-killing bacterial kinetic assay of the methanolic leaf extract against the *E. coli* clinical case. 1/2MIC = 0.31 mg/mL; MIC = 0.62 mg/mL; MBC = 1.25 mg/mL.

Based on the above results, the bacteria and yeast most sensitive to the methanolic leaf extract were tested in a time-killing kinetic assay to observe whether the extract had a fungi/bacteriostatic or fungi/bactericidal effect. In the case of the bacterial strains, *S. aureus* ATCC 12,398 and *E. coli* (clinical case) were tested. The methanolic extract displayed a bacteriostatic effect against *S. aureus* ATCC 12,398, showing similar behaviors at the three tested concentrations, whereas in the case of *E. coli*, although the extract also showed a bacteriostatic effect, the MBC had the greatest activity (Figs. 3 and 4).

**Figure 5:**
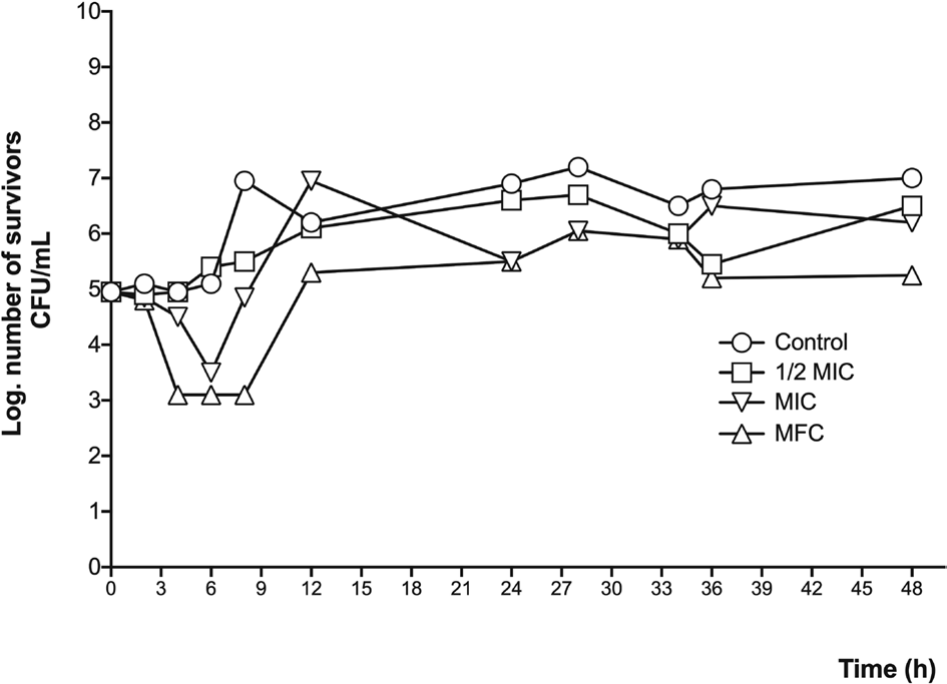
Time-killing fungal kinetic assay of the methanolic leaf extract against the *C. tropicalis* clinical case. 1/2 MIC = 0.078 mg/mL; MIC = 0.156 mg/mL; MFC = 0.62 mg/mL.

**Figure 6:**
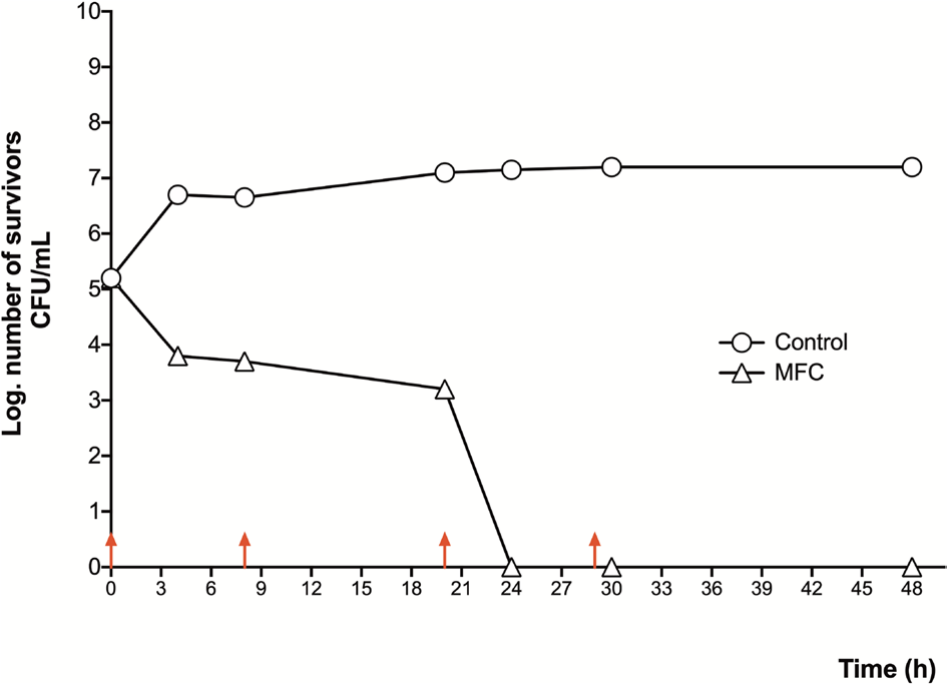
Time-killing fungal kinetic assay of the methanolic leaf extract against the *C. tropicalis* clinical case. MFC = 1.24 mg/mL. Red lines indicate the times when the extract was added to the culture.

In the case of *Candida* species, the methanolic extract showed a fungistatic effect on all four strains used (*C. albicans* cc, *C. albicans* ATCC 10231, *C. glabrata* cc and *C. tropicalis* cc). However, after observing the activity of the methanolic extract on *C. tropicalis* exposed to the MFC (Fig. 5), it was decided added three times this concentration in a new test, which produced fungicidal activity (Fig. 6).

## Discussion

The main secondary metabolite groups that have been reported to be responsible for the antimicrobial activity of the plants from the genus *Prosopis* are phenolic compounds and alkaloids^8–10^. The contents of phenolic compounds and flavonoids were, in general, less than those reported by previous studies on the phytochemical composition of the leaves and flowers from the pods obtained from several *Prosopis* species^8–10^; however, and even though the compounds detected through GC–MS and HPLC–MS had no matches with the available libraries, the partial results obtained by HPLC–DAD in the present study confirm the widespread presence of some simple phenols and catechol, a compound common in various plant species^14–16^. In the case of the alkaloids, these were in the range previously reported for various species of *Prosopis*^15–20^. Alkaloids, together with flavonoids, have been reported to be mainly responsible for the antimicrobial activity in this genus^8–10,17,19^. However, more studies are needed regarding the composition of the methanolic extract to determine its complete phytochemical profile.

The antioxidant activity of *Prosopis spp.* has been studied for several species and attributed to alkaloids and phenolic compounds present in the aerial parts of *P. juliflora* and *P. laevigata*^20–22^. However, in the present study, the obtained value (AC_50_ = 334.78 ppm) is higher than that found in other studies^18–21^ and could be related to the composition of the methanolic extract, probably due to the low concentration of phenolic compounds in comparison with previous studies^13,14,21,22^.

The antibacterial activity of the methanolic extract is comparable to the results of Sanchez et al.^23^, whose research showed that the antibacterial activity of the methanolic extract of the aerial parts of *Prosopis laevigata* was higher than that of other methanolic extracts from other medicinal species. On the other hand, the studies by Salinas et al.^24^, Khan et al.^25^, and Valli et al.^19^ presented inhibitory concentrations of different solvent extracts from others *Prosopis* species that were less than 100 µg/mL. These activities could be related with the alkaloid fraction in the extracts, as several studies reported that characteristic alkaloids from this genus (like prosopine, cassine, prosoflorine and juliprosopine) had shown alterations to the plasma membrane permeability either by intercalating between the phospholipids that make it up or affecting the functions of some ion channels, thus provoking cell lysis^17–20^. In our study, the methanolic leaf extract showed important antibacterial activity, although it was less effective than the studies mentioned above. However, the measured activity against medically interesting species, such as *S. aureus* and *E. coli*, presented in this study reveals the potential of the chemical constituents of the methanolic extract, as they had a bacteriostatic effect.

In the case of fungicidal activity, the results obtained with *Candida* species are comparable with the results obtained by Khan et al.^25^, whose research focused on the activity of several extracts from *P. spicigera* against multirresistant strains, and Jahromi et al.^14^, with an ethanolic extract from *P. farcta* against some *Candida albicans* strains. On the other hand, the activity in the fungal growth kinetic assay, especially for *C. tropicalis*, opens the possibility of using the compounds from the methanolic extract, either in isolation or in standardized mixtures, in studies to identify their ability to treat some forms of candidiasis.

On the other hand, our methanolic leaf extract showed results against filamentous fungi from the *Fusarium* genus that were better than those from other studies that used extracts from several parts of different *Prosopis* species against filamentous strains with high agronomic importance, such as *Alternata alternata, Rhizopus stolonifera, Aspergillus fumigates* and *Lasiodiploida theobromae*^25–27^. Most of these studies reported minimal fungicidal concentrations under 2 mg/mL, which are lower than those reported in the present study, all of which were above 4 mg/mL. The effect on different fungal strains could be related to the contained alkaloids and phenylpropanoids, which have important for ion regulation and cell wall and membrane functions and could produce important damage to these organelles^16,18,19^.

Based on the observed results, it can be concluded that the *Prosopis laevigata* methanolic leaf extract has a strong antimicrobial activity, especially against *Candida* strains; it also, has bacteriostatic activity against medically critical bacterial species such as *S. aureus* and *E. coli*, and it has fungistatic activity with a single application at a single time, and fungicidal activity when applied at different times over *Candida tropicalis* and an important activity over *Fusarium spp*. These effects could be related to the chemical composition of the extract, which is currently being studied.

## Methods

### Collection of plant material

The leaves of *P. laevigata* were collected in Zapotitlan Salinas, in the Tehuacán-Cuicatlán Valley, between 18° 19′ 46ʺ N and 97° 30′ 58ʺ W at an altitude of 1553 m above sea level in March 2018. Voucher specimens were identified by Biol. María Edith López Villafranco and deposited in the IZTA herbarium (IZTA 3223). *P. laevigata* is not endangered. The specimens were collected in the field with permission from Secretaría de Medio Ambiente y Recursos Naturales (SGPA/DGVS/1266). The study of this species complied with relevant institutional, national, and international guidelines and legislation.

### Obtention of the methanolic extract

The leaflets were separated from the branches and dried at room temperature for 7 days. The dried plant material (75.9 g) was powdered and dissolved in methanol. After the solution was filtered through No. 1 filter paper and distilled under vacuum at 50 °C to remove the methanol, 18.216 g (24.0%) of crude material was left.

### Detection of secondary metabolites

For each test, 5 mg of the leaf extract was weighted.

The presence of phenolic compounds was detected, dissolving the sample in 3 mL of water and adding two drops of 5% ferric chloride solution. The test was considered positive if the color changed to dark blue^28^.

The presence of flavonoids was detected dissolving the sample in 3 mL of methanol and adding two drops of a 2% aluminum chloride solution. The test was considered positive if the color changed to bright yellow^28^.

The presence of alkaloids was detected by dissolving the sample in 3 mL of water and acidifying the solution with a 1 N hydrochloric acid solution; then, 2 or 3 drops of Dragendorff’s reagent were added. The test was considered positive when a reddish-colored precipitate formed^29^.

### Quantification of secondary metabolites

#### Total phenolic compounds content

The concentration of total phenolic compounds was determined by the Folic-Ciocalteau method. A standard curve in a range of 6.25 to 200 µg of gallic acid was made in assay tubes, and a tube problem with a volume of 1200 µL of a solution of 200 µg/mL methanolic leaf extract; all tubes were gauged to 8 mL with distilled water. Then, 500 µL of Folin–Ciocalteau reactive were added, and, after 5 min, 1500µL of a 200 g/L Na_2_CO_3_ solution were added. The standard curve and problem were left to stand at room temperature in the dark for two hours, and the absorbance was measured at 760 nm. The results were reported as mg EGA/mL^30^.

#### Total flavonoids content

The concentration of total flavonoids was determined by the Dowd method. A standard curve in a range of 1 to 1000 ppm of quercetin was made in assay tubes, and a tube problem with a volume of 1000µL of a 1 mg/mL solution methanolic leaf extract. All volumes were gauged to 1000 µL with HPLC grade methanol; then, 1000 µL of 2% aluminum chloride were added. After 5 min, 200 µL of each tube were deposited in a 96-well plate for triplicate and the plate absorbance was measured at 450 nm wavelength. The results were reported as µg QE/mL^31^.

#### Total alkaloids content

The concentration of total alkaloids was determined by the bromocresol green method. A standard curve in a range of 20 to 100 µg of atropine was made in assay tubes and a tube problem with a volume of 1200µL of a 2 mg/mL of methanolic leaf extract. 2500 µL of a 2 M sodium phosphate at a pH of 4.7 were added to each tube; then, 2500 µL of bromocresol green were added, and each tube were washed with 5 mL of chloroform; the chloroform was recovered, and the absorbance was measured at 470 nm wavelength. The results were reported as mg EA/mL^32^.

### Antioxidant capacity

The antioxidant capacity was determined by the 2,2-diphenyl-1-1-picrylhydrazyl method^13^. A series of concentrations of the methanolic extract of leaves was made in a range of 1 to 1000 ppm from a stock of 1 mg/mL of leaf methanolic extract in assay tubes and gauged to 1 mL with HPLC grade methanol. After that, 50 µL of each concentration were deposited for triplicated in a 96-well plate, and then 150 µL of 100 μM DPPH solution were added. The plate was incubated at 37 °C for 30 min; the absorbance was then measured at a wavelength of 540 nm. To determine the reduction of DPPH, the following formula was applied:

$$\text{\% of DPPH reduction}=\frac{\text{C}-\text{E}}{\text{C}} \times 100$$

C = DPPH absorbance; E = DPPH + leaf methanolic extract absorbance.

With those results, a standard curve was made, and the medium antioxidant capacity (AC_50_) was calculated and reported in ppm^13^.

### Chemical analysis of the methanolic extract

The methanolic leaf extract was analyzed by GC‒MS, HPLC–DAD, and HPLC‒MS.

For GC‒MS analysis, a model 6850 chromatograph (Agilent Technologies, Santa Clara, CA, USA) coupled with a model 5975C mass spectrometer (Agilent Technologies) and an HP-5MS column (30 m × 0.25 mm, 0.25 µm; Agilent Technologies) was used. For the sample, 1 µL of extract from a 1 mg/mL solution was dissolved in HPLC-grade methanol and injected in split mode. The separation conditions were as follows: an initial temperature of 70 °C for 2 min followed by two increases: the first was 20 °C/min to 230 °C, and the second was 15 °C/min up to 290 °C, which was then maintained for 1 min. Helium was used as the carrier gas. The total analysis time was 26.67 min. The detected mass range was 35–600 m*/z*, the sample was ionized by electron impact at 70 eV, and the ionization source temperature was 230 °C. To identify the compounds, the NIST database was used.

For HPLC–DAD analysis, a Hewlett-Packard HP series 1100 (Hewlett-Packard, Wilmington DE, USA) instrument operated with ChemStation A0903 software and a Discovery C18 column (250 × 4.6 mm, particle size of 5 µm) was used. The sample was eluted with an isocratic mixture of methanol:acetonitrile:water (25:25:50) with a flow rate of 1 mL/min and measured at a wavelength of 260 nm with a complete screen from 200 to 400 nm.

HPLC‒MS analysis was performed using an Agilent 1200 Infinity LC coupled to an Agilent 6230 TOF mass spectrometer with an Agilent Dual ESI source (ESE SG14289023) and MassHunter Workstation software, version B.05.01, build 5.01.5125.3, operating in negative ionization mode. The capillary voltage was 4500 V; the dry gas temperature was 250 °C; nitrogen was used as the drying gas at a flow rate of 6 L/min; the nebulizer pressure was 60 psig; the fragmentor voltage was 200 V; the MS range was 50–1500 m*/z*; and the MS acquisition rate was 1 spectrum/s. Chromatographic separation was accomplished using an HPLC system (Infinity Series 1200, Agilent Technologies, Waldbronn, Germany) equipped with a Kinetex 2.6 u, C18 100A column (150 × 2.1 mm) (Phenomenex, SA, Torrance, CA, USA). The column temperature was maintained at 25 °C. The following gradient program was used along with a two-component mobile phase consisting of water:acetonitrile (90:10) with 1.0% formic acid (solvent A) and methanol:acetonitrile (90:10) with 1.0% formic acid (solvent B). The initial conditions were 3 min with 100% solvent A, followed by 3–5 min: 65% A; 5–15 min: 50% A; 15–30 min: 0% A; and 40 min: 0% A.

The following HPLC database standards were used: kaempferol, catechin, pinocembrin, baicalein, naringin, naringin, catechol, quercetin, luteolin, genistein, caffeine, apigenin, myricetin, chrysin, and acacetin. All standards were purchased from Sigma‒Aldrich (St. Louis, USA).

### Test microorganisms

#### Bacterial strains

*Staphylococcus aureus* ATCC 12,398 (resistant to ampicillin, penicillin, cefotaxime, cefuroxime, and dicloxacillin), *Staphylococcus aureus* clinical case donated by “Clinica Universitaria de Salud Integral Iztacala UNAM” (CUSI-IZTA) (resistant to ampicillin, erythromycin, penicillin, cefotaxime, cefuroxime and dicloxacillin), *Staphylococcus epidermidis* ATCC 12,228 (resistant to ampicillin, erythromycin, penicillin, cefotaxime, cefuroxime and dicloxacillin), *Staphylococcus epidermidis* clinical case donated by CUSI-IZTA (resistant to ampicillin, erythromycin, penicillin, cefotaxime, and dicloxacillin), *Enterococcus faecalis* ATCC 29,210 (resistant to ampicillin, erythromycin, penicillin, cefotaxime, cefuroxime and dicloxacillin), *Pseudomonas aeruginosa* CDBBB999 (resistant to cefepime, cefotaxime, and ceftriaxone), *Escherichia coli* ATCC 25,922 (resistant to cefepime, cephalothin), *Escherichia coli* clinical case^1^ donated by CUSI-IZTA (resistant to cefepime, cephalothin, ampicillin and ceftriaxone) *Escherichia coli* clinical case^2^ donated by CUSI-IZTA (resistant to cephalothin and ampicillin) and *Vibrio cholerae* clinical case donated by Laboratory of Microbiology of FES-Cuautitlan UNAM (FES-C) (resistant to cefepime, cefotaxime and ceftriaxone) were used. The antibiotic resistance of all strains was determined by Bio-Rad ® SA multidisc to Gram positive II and Gram negative antibiograms (Mexico City, Mexico).

#### Yeast fungal strains

*Candida albicans* ATCC 14,065, *Candida albicans* ATCC 32,354 (resistant to fluconazole and ketoconazole), *Candida albicans* CDBB-L-1003 (resistant to fluconazole and ketoconazole), *Candida albicans* ATCC 10,231 (resistant to fluconazole), *Candida albicans*, clinical case^1^ donated (FES-C) (resistant to fluconazole), *Candida albicans,* clinical case^2^ donated by Hospital Los Angeles (resistant to fluconazole), *Candida tropicalis* CDBB-L-1098 (resistant to fluconazole), *Candida tropicalis* clinical case^1^ donated by Hospital Los Angeles (resistant to fluconazole), *Candida tropicalis,* clinical case^2^ donated (FES-C) (resistant to fluconazole), *Candida tropicalis* clinical case^3^ donated by Hospital Los Angeles (resistant to fluconazole) *Candida tropicalis,* clinical case^4^ donated by Hospital Los Angeles (resistant to fluconazole), *Candida glabrata* CBS 138 (resistant to fluconazole), *Candida glabrata* CDBB-L1536 (resistant to fluconazole), *Candida glabrata,* clinical case^1^ donated by CUSI-IZTA (resistant to fluconazole) and *Candida glabrata* clinical case^2^ donated (FES-C) (resistant to fluconazole) were used. The antibiotic resistance of all strains was determined by Bio-Rad ® antifungigram discs for azoles, nystatin, amphotericin B and imidazoles (Mexico City, Mexico).

#### Filamentous fungal strains

*Fusarium moniliforme* CDBB-H-265, *Fusarium subglutinans, Fusarium sporotrichioides* ATCC NRLL3299*, Fusarium oxysporum, Rhizoctonia lilacina* CDBB-H-306 were used*.*

#### Antibacterial activity assay

Antibacterial activity was evaluated using the Kirby-Baüer disk diffusion agar method^33^. Bacteria were cultured in Müeller-Hinton broth at 36 °C for 24 h. The density of the cultures was adjusted to a turbidity comparable to McFarland standard no. 0.5 with sterile saline solution (1.5 × 10^8^ CFU/ml) and were inoculated evenly over the surface of Petri dishes with 30 mL of Müller-Hinton agar (Bioxon, Estado de Mexico, Mexico). Five-millimeter diameter disks (Whatman no. 5) impregnated with 2 mg of methanolic extract were placed on the surface of the inoculated plate, and disks with 25 µg of chloramphenicol were used as positive controls. The Petri dishes were incubated for 24 h. The diameters of the clear zones around the discs were measured using calipers. The tests were performed in triplicate.

A broth microdilution assay^34^ was used to evaluate the minimum inhibitory concentration (MIC) and bactericidal minimum concentration (BMC). The concentrations were in the range of 0.312–20 mg/mL. Microtubes were inoculated with a 10^5^ CFU/mL bacterial suspension. Inoculated plates were incubated at 36 °C for 24 h. After incubation, the plates were exposed to 0.08% tetrazolium chloride. A negative control was not necessary because culture broth was used as the solvent for the methanolic extract stock solution. Each experiment was repeated at least three times. To determine the MIC and BMC, the plates were observed, comparing the size of the bacterial point under leaf extract concentrations with the control. The concentration in which a decrease in the size of the bacterial button was found to that of the control was declared as MIC, and aliquots were taken from the higher concentrations to be cultivated in Petri dishes, which were incubated for 24 h at 37 °C, after which the colonies were counted. The lowest concentration in which no bacterial growth was declared MBC.

The effect on bacterial growth was determined with a time-killing bacterial kinetic assay; for that purpose, cultures with a concentration of 1 × 10^5^ colony forming units (CFU) were made; the groups were control and three cultures with the concentration of 1/2 MIC, MIC, and MBC; culture growth was monitored at eight times (4 at 1-h intervals, 3 at 2-h intervals, one after 12 h and one after 24 h); At each time a sample was taken and planted in a petri dish with three septa; to facilitate counting, dilutions of 1:100 and 1:10,000 were made in physiological solution, which was also seeded in septated plates and incubated for 24 h; After this, the CFU were counted, taking into account the dilution adjustment and they were plotted on base 10 logarithm^35^.

#### Antifungal activity assay

Antifungal activity was determined with diffusion agar assays^33^. Yeast inoculum was incubated in 10 ml of Sabouraud broth at 36 °C for 24 h. The cultures were adjusted to a turbidity comparable to McFarland standard no. 0.5 with sterile saline solution (1 × 10^6^ CFU/mL). The yeast suspensions were inoculated evenly over the surface of Petri dishes with 30 mL of potato dextrose agar (Bioxon, Estado de Mexico, Mexico). Five-millimeter diameter disks (Whatman no. 5) were impregnated with 4 mg of methanolic extract, and 25 µg of nystatin was used as a positive control. The Petri dishes were incubated for 48 h at 36 °C. The diameters of the clear zones around the methanolic extract-impregnated disks were measured using calipers. The tests were performed in triplicate.

A broth microdilution assay^34^ was used to evaluate the 50% medium inhibitory concentration (MIC) and minimum fungicidal concentration (MFC). The concentrations were in the range of 0.625–40 mg/mL. Microtubes were inoculated with a 10^5^ CFU/mL yeast suspension. Inoculated plates were incubated at 36 °C for 24 h. After incubation, the size of the visible fungal point was observed and compared with the size of the control. Negative control was not necessary because culture broth was used as the solvent for the methanolic extract stock solution solvent. Each experiment was repeated at least three times. To determine the MIC and MFC, the plates were observed, comparing the size of the yeast point under leaf extract concentrations with the control. The concentration in which a decrease in the size of the yeast button was found with respect to that of the control was declared as MIC, and aliquots were taken from the higher concentrations to be cultivated in Petri dishes, which were incubated for 24 h at 37 °C, after which the colonies were counted. The lowest concentration in which no yeast growth was declared MFC.

The effect on fungal growth was determined with a time-killing fungal kinetic assay; for that purpose, cultures with a concentration of 1 × 10^5^ colony forming units (CFU) were made; the groups were, control and three cultures with the concentration of 1/2 MIC, MIC, and MBC; culture growth was monitored eight times (4 at 2-h intervals, one at 4-h intervals, and one at 12-h interval, three at 4-h intervals, and one at 12-h interval); At each time, a sample was taken and planted in a petri dish with three septa; to facilitate counting, dilutions of 1:100 and 1:10,000 were made in physiological solution, which were also seeded in septated plates and incubated for 24 h; After this, the CFU were counted, taking into account the dilution adjustment and they were plotted on base 10 logarithm^35^.

The antifungal activity against filamentous fungi was determined by the radial growth inhibition method^36^. Petri dishes with PDA were inoculated with 5 mm diameter mycelium and incubated at 28 °C for 72 h. Once the mycelium had developed, paper filter discs with 4 mg of methanolic extract were placed over these plates. A positive control disc with 7.0 µg of ketoconazole was used. Mycelium reduction was reported as antifungal activity. The tests were performed in triplicate.

The CF_50_ values against the filamentous fungi were determined by agar dilution assays^34^ in 24-well culture plates with 1 mL of potato-dextrose agar, and a range of concentrations from 0.25 to 8 mg/mL methanolic extract were used. When the agar solidified, a mycelium button 1 mm in diameter was placed in the middle of the surface of each well. Each day, the culture dishes were monitored until the surface of the controls were covered with the fungal strain used. The diameter of mycelial growth in the control wells was measured and used to determine the mean fungicidal concentration. A negative control was not necessary because culture broth was used as the solvent for the methanolic extract stock solution. Radial growth inhibition was calculated with the formula RGI = (15–×) • 100, where 15 is the total diameter of the well and x is the diameter of mycelial growth. The test was performed in triplicate.

### Statistical analysis

The mean and standard deviation of the experiments were determined. Analysis of variance (ANOVA) was performed to test for significant differences (*p* < 0.05) with Tukey’s honestly significant difference (HSD) multiple comparison test using the GraphPad Prism version 7 program.

## Data Availability

All data generated or analyzed during this study are included in this published article.

## Abbreviations

HPLC–DAD: Diode array high performance liquid chromatography
GC–MS: Gas chromatography coupled to mass spectrometry
HPLC–MS: High performance liquid chromatography coupled to mass spectrometry
MIC: Minimal inhibitory concentration
MBC: Minimal bactericide concentration
MFC: Minimal fungicide concentration
